# *In Vitro* and *In Vivo* Osteogenesis Induced by Icariin and Bone Morphogenetic Protein-2: A Dynamic Observation

**DOI:** 10.3389/fphar.2020.01058

**Published:** 2020-07-14

**Authors:** Lina Xie, Ning Liu, Ye Xiao, Yanhui Liu, Chunge Yan, Gailing Wang, Xiangdong Jing

**Affiliations:** ^1^Department of Stomatology, The First Affiliated Hospital of Guangzhou University of Chinese Medicine, Guangzhou, China; ^2^School of Life Sciences, Hunan Normal University, Changsha, China

**Keywords:** icariin, bone morphogenetic protein-2, traditional Chinese medicine, osteogenesis, bone repair, nano-hydroxyapatite/collagen/polylactic acid (nHAC/PLA), dyeing method

## Abstract

In the present study, we aimed to compare the effects of icariin (ICA) and bone morphogenetic protein-2 (BMP-2) on osteoblast proliferation and osteogenesis in bone defects. We found that *in vitro* ICA or BMP-2 treatment is able to increase osteoblast proliferation, which was detected by 3-(4,5-dimethylthiazol-2-yl)-2,5-diphenyltetrazolium bromide (MTT). Specifically, ICA at a concentration of 30 µg/ml had the strongest ability to promote cell proliferation, which is equivalent with the effect of BMP-2 at a concentration of 50 µg/ml. Furthermore, Western blot and RT-qPCR analyses showed that treatment with ICA (20–30 µg/ml) had similar increase effect with BMP-2 (50 µg/ml) on the protein and mRNA levels of *BMP-2*, osteoprotegerin (*OPG*), and alkaline phosphatase (*ALP)* mRNAs. In addition, the animal model of bone defects was successfully prepared. The *in vivo* data showed that compared with the control group, highest osteogenesis in the ICA or BMP-2 groups was observed at different observational times. Four weeks after surgery, osteogenesis in the BMP-2 group was slightly higher than that in the ICA group, but there was no significant difference between the two groups until the eighth week. ICA promotes osteoblast proliferation by stimulating the expression of BMP-2 and OPG proteins and upregulating the expression of BMP-2, OPG, and ALP mRNAs. ICA at a certain concentration has the same osteogenic effect as BMP-2. ICA or BMP-2 composite nanomaterials can be used as a framework to guide bone regeneration and promote osteogenesis. In addition, the combined use of hematoxylin-eosin and Goldner’s trichrome staining techniques contributes to acquiring better bone morphometric information about bone defects.

## Introduction

The shape of a defect in the jaw caused by tumor, trauma, inflammation, and periodontitis is often irregular, which seriously affects chewing, pronunciation, and maxillofacial appearance. Bone remodeling in the defect area has always been an issue that besets clinicians. With the development of tissue engineering and implementation of traditional Chinese medicine, the use of bone scaffold composites is an effective method to improve the effect of transplantation using bone xenograft. Recent findings both *in vivo* and *in vitro* suggest that natural Chinese medicine may provide potential therapeutic benefits for treatments in tandem with both bone formation and anti-resorption effects. The combination of active ingredients of Chinese herbs and bioactive materials may be a concern in bone defect repair (Han et al., 2017; He et al., 2019; Shi et al., 2019). Icariin (ICA) is a major active ingredient of *Epimedium brevicornum* Maxim and exerts diverse bioactivities (Zhang et al., 2014; Liu et al., 2019; Zi et al., 2019). Bone morphogenetic proteins (BMPs) are the only cytokine family found to induce ectopic osteogenesis. BMP-2 is a well-recognized cytokine with strongest osteogenic activity (Salazar et al., 2016). Previous studies have reported little on the promotion of osteogenesis by a combination of ICA or BMP. Domestic studies have shown that with the addition of BioCaP granules, co-administration of ICA and BMP-2 was helpful in bone tissue engineering through histological and histomorphometric ﬁndings in the critical sized bone defects of Sprague Dawley rats (Zhang et al., 2019). Our previous studies have confirmed the role of periosteum and nano-hydroxyapatite/collagen/polylactic acid (nHAC/PLA) in bone repair (Xie et al., 2010). To date, the combined use of nHAC/PLA with Chinese herb monomer has not been reported in animal models or clinical practice. In this study, hFOB1.19 osteoblasts, highly homological to human osteoblasts, were cultured with ICA or BMP-2 to compare the effects of these interventions on osteoblast proliferation and osteogenesis in bone defects, and their growth characteristics were assessed.

## Materials and Methods

### Materials, Reagents, and Main Instruments

ICA (≥94%; Sigma-Aldrich, St. Louis, Missouri, USA), BMP-2 (≥98%; Sigma-Aldrich), hFOB1.19 osteoblasts (Cell Bank, Chinese Academy of Sciences, Shanghai, China), DMSO (Sigma-Aldrich), MTT solution (Beijing, Beijing Solarbio Science & Technology Co., Ltd, China), Western Blot test reagent (Thermo Scientific, Bartlesville, OK, USA), Tanon™ High-sig ECL Western blotting Substrate (Thermo Scientific), polyvinylidene fluoride (PVDF) membrane (Millipore, Etobicoke, ON, Canada), SDS (Shanghai, Shanghai ruji biotechnology development co. LTD, China), SuperScript IV reverse transcriptase (Thermo Scientific), and dyeing liquid (Shanghai, China) were used in the study. Eppendorf BioPhotometer D30 (Eppendorf, Hamburg, Germany) and Tanon 5200 automatic chemiluminescence image analysis system (Tanon Science & Technology Co. Ltd., Shanghai, China) were used in this study.

### MTT Assay

ICA mother liquor was dissolved to 1 μg/ml by 0.01% dimethyl sulfoxide (DMSO), and stored at 4°C until further use. hFOB1.19 cell suspension (2 × 10^4^ cells/ml) as passage 3 osteoblasts were prepared and seeded into a 96-well culture plate at a density of 100 μl per well with four replicate wells in each group. The cells were cultured at 37°C and 5% CO_2_. The next day, the cells were cultured in another media containing different concentrations of ICA or BMP-2 and incubated for 48 h continuously and MTT (10 μl per well, 5 mg/ml) was added to each well at the 44th hour. The supernatant in each well was carefully aspirated after another 4 h, and 100 μl of DMSO was added to each well. The samples were shaken for 10 min and absorbance was measured at 490 nm with a microplate reader. The measured values in each group were statistically analyzed.

### Western Blot Assay

After 72 h of culture, cells of each group were collected, and cell lysates were obtained, placed on ice for 30 min, boiled in a 100°C water bath for 10 min, iced for 10 min, and centrifuged at 64,000 × *g* for 5 min. The supernatant was quantitatively measured using a UV spectrophotometer and then stored at −20°C until further use. The sample in each group was electrophoresed on 10% sodium dodecyl sulfate-polyacrylamide gel electrophoresis (SDS-PAGE), and transferred onto a PVDF membrane. Following blocking with 50 g/L skim milk for 1 hour, the sample was rinsed, incubated with the primary antibody (antiserum of the target protein) (1:200) at 4°C overnight, and further incubated with the secondary antibody (HRP, horse radish peroxidase) (1:100) for 2 h at 24°C. The sample was developed using the electrochemiluminescence (ECL) method, with β-actin as an internal reference. Expression of OPG and BMP-2 was compared based on the width of each band.

### Quantitative Real-Time PCR

Total RNA was extracted and the integrity of RNA was determined. Bands of 28S and 18S were scanned using a laser density scanner, and converted into cDNA with a reverse transcription kit. A quantitative real-time PCR (qPCR) system was prepared in a dedicated 96-well plate. One microliter of cDNA was used to amplify *OPG*, *BMP-2*, and *ALP* genes. Primer sequences and amplification conditions are shown in **Table 1**. Cycle threshold (Ct value) of the target gene and internal reference and relative quantification (RQ) value were determined (RQ = CT^target^/CT^sample^). Higher values indicate greater expression.

**Table 1:** Primer sequence and amplification conditions for real-time qPCR.

Gene	Primer sequences (5′-3′)	Accession no.	Product size (bp)
OPG	GTGTGCGAATGCAAGGAAGG	NM__002546	82
	CCACTCCAAATCCAGGAGGG		
ALP	CCCGCTTTAACCAGTGCAAC	NM__001632	224
	GAGCTGCGTAGCGATGTCC		
BMP-2	ACCCGCTGTCTTCTAGCTAT	NM__001200	180
	TTCAGGCCGAACATGCTGAG		
β-actin	CGTGGACATCCGCAAAGAC	KJ896369.1	234
	TCGTCATCCTCCTGCTTGCTG		

### Materials, Reagents, and Instruments

nHAC/PLA was provided by Tsinghua University Materials Department, China. Electronic balance, microscope, slicer, LEICA2500E non-decalcification slicer, and tungsten steel slicer were provided by the People’s Liberation Army General Hospital, China. DIA-2000 pathological image analyzer was provided by Wuhan University, China. Hematoxylin-eosin (HE) stain and Goldner’s trichrome stains were purchased from Sigma-Aldrich.

### Animals Models

Eighteen healthy female New Zealand white rabbits (Institute of Laboratory Animals, Chinese Academy of Agricultural Sciences, China), 6 months of age, were randomly divided into three groups: control, ICA, and BMP. The animals were numbered and caged with mixed feed for one week. The animal experiments were performed strictly in accordance with the regulations of Guangdong Experimental Animal Center, China. The Experimental Animal Ethics Committee of Guangzhou University of Chinese medicine, China approved and reviewed the experimental animal program, which conforms with the animal protection, animal welfare and ethical principles, and the relevant provisions of the national experimental animal welfare ethics.

On the first- and second-day post-operation, all the rabbits had poor appetite and were less active than before. However, they showed no signs of cold and fever, had no abnormal secretions in the surgical area, and had no rashes on the whole body. After 3 days, their activities returned to normal. They were then euthanized and enrolled for analysis.

**Figure 1:**
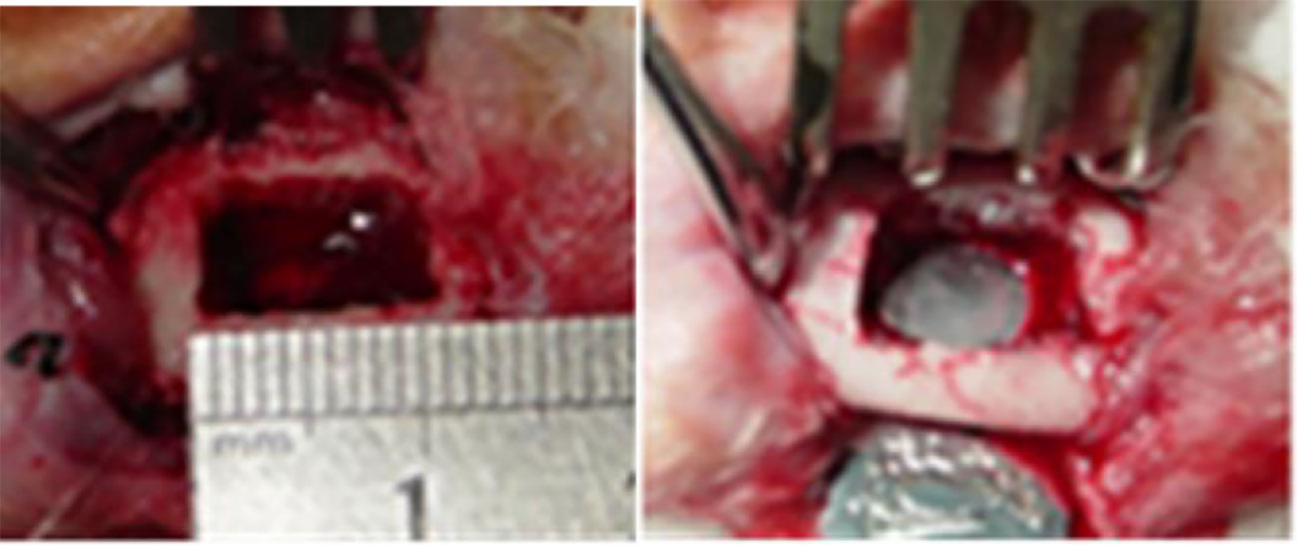
Preparation of a rabbit model with mandibular defect.

An animal model with full-thickness rectangular bone defect in the periosteum, 10 mm × 8 mm, was prepared in the right mandible of a rabbit. No intervention was done in the control group. Rabbits in the ICA or BMP groups were implanted with 30 μg/ml ICA/nHAC/PLA and 50 μg/ml BMP-2/nHAC/PLA, respectively (**Figure 1**). We introduced ICA or BMP-2 into the pores of nHAC/PLA materials through external vacuum adsorption. Two experimental animals from each group were euthanized at each time point (4, 8, and 12 weeks post-operation).

### Histological Observation

Tissues with bone defects were segmented, and buccolingually cut into two halves. Bone block at the buccal side was cut into non-decalcified sections for Goldner’s trichrome staining, while bone block at the lingual side was cut into decalcified sections for HE staining.

### HE Staining

The defective tissue block was fixed, decalcified, dehydrated, transparentized, embedded, and sectioned, after baking, dewaxing, and staining by the following steps: hematoxylin soaking for 15 to 20 min, water flushing for 1 min, 1% hydrochloric acid ethanol differentiation for 5 s, washing with water for 15 min (color change from black to blue) and distilled water for 1 to 2 min, and immersed in eosin for 10 to 12 min. It was then dehydrated and transparentized. This was followed by dropping gum on the transparent slice and covering the glass slide to seal it. The morphology of the tissue was observed under an optical microscope.

### Goldner’s Trichrome Staining

The tissue block was fixed and sliced at a thickness of 4 μm with a Leica 2500E non-decalcification slicer and a tungsten slicer. The sections were placed in a 42°C oven overnight and stained the next day. The staining steps were: The sections were (1) stained with Weigert’s iron hematoxylin solution for 15 min, rinsed with distilled water, washed with running water for 15 min, and rinsed with distilled water; (2) the sections were then stained with ponceau acid fuchsin solution for 15 min, and rinsed twice using 1% acetic acid; (3) the sections were further stained in phosphotungstic acid-orange G for 8 min and rinsed twice with 1% acetic acid; (4) the sections were stained in light green SF yellowish for 15 min and rinsed twice with 1% acetic acid; and (5) the sections were finally rinsed with 70% ethanol once, 100% ethanol-dehydrated twice, transparentized in xylene, mounted with resin, and observed under a microscope.

### Statistical Analyses

Statistical analyses between multiple groups were analyzed by one-way ANOVA followed by Tukey *post hoc* test using GraphPad Prism 7.6.1 (GraphPad Software Inc., San Diego, CA, USA). Quantitative data for all data were expressed as mean ± standard deviation (SD). Values with *P* <0.05 were considered statistically significant, and P <0.01 were considered very statistically significant.

## Results

### ICA Promotes Osteoblast Proliferation

To investigate the effect of ICA on the osteoblast proliferation, hFOB1.19 cells were treated with dose-dependent ICA or BMP2, which was used as a positive control. The results showed that treatment with 10 to 40 μg/ml of ICA and 50 μg/ml BMP-2 significantly promotes osteoblast proliferation after 48 h of culture (*P <*0.05 vs. control), although treatment with ICA with the concentration of 50 μg/ml has no significant effect (**Table 2**). These results suggested that ICA plays a role in promoting osteoblast proliferation.

**Table 2:** MTT detection of cell proliferation after intervention with icariin (ICA) and bone morphogenetic protein-2 (BMP-2) at different concentrations.

Group	Cell proliferation (A490 nm; 48 h)
Control	0.1310.013
10μg/mL ICA	0.171± 0.026*
20 μg/mL ICA	0.183±0.023*
30 μg/mL ICA	0.253±0.016**
40 μg/mL ICA	0.206±0.015*
50 μg/mL ICA	0.165±0.008
50 μg/mL BMP-2	0.254±0.016**

The same day compared with control group; *P < 0.05, **P < 0.01.

### ICA Increases BMP2 and OPG Protein Levels

**Figure 2:**
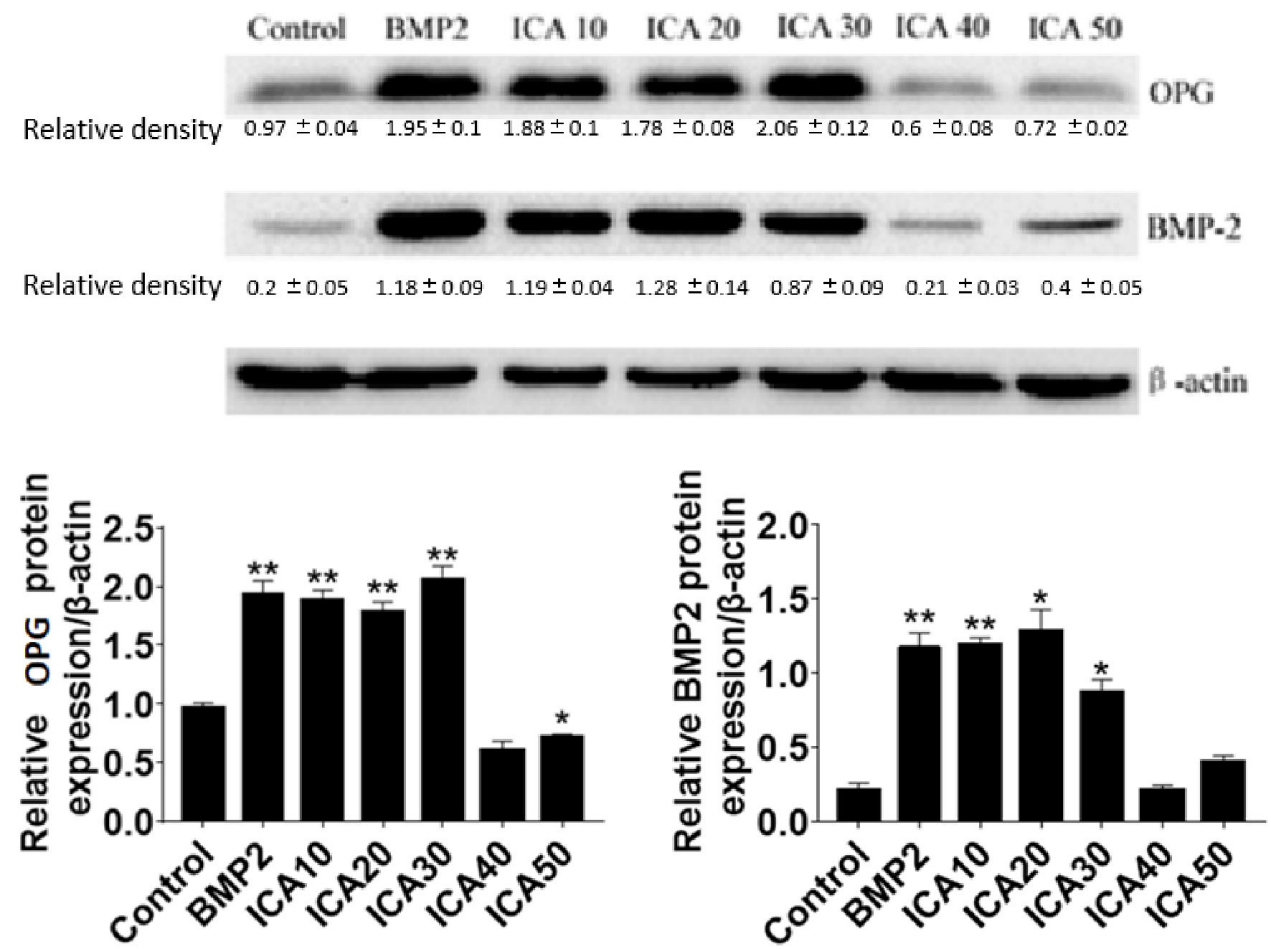
Western blot detection of BMP-2 and OPG protein expression after Q11 intervention with different concentrations of ICA and BMP-2. *p < 0.5, **p < 0.1.

To investigate the mechanisms underlying the role of ICA in promoting osteoblast proliferation, the expression of two important proteins include BMP2 and OPG, which are involved in regulating osteoclastogenesis and osteoclast differentiation were determined. The results showed that the protein levels of BMP-2 and OPG were significantly increased after treatment with 10 to 30 μg/ml ICA or 50 μg/ml BMP-2 for 48 h compared with the control group. However, we observed that 40 and 50 μg/ml ICA could not further increase the expression of BMP-2 and OPG. These results suggested that ICA is able to increases BMP2 and OPG protein levels (**Figure 2**).

### ICA Increases the mRNA Levels of *BMP2, OPG*, and *ALP* Genes

**Figure 3:**
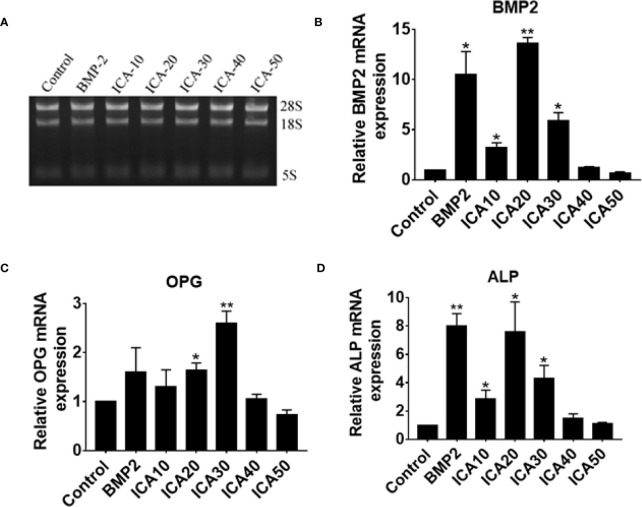
**(A)** Extraction of total RNA and determination of RNA integrity via gel electrophoresis and qPCR detection of relative expression of **(B)** BMP-2, **(C)** OPG, and **(D)** ALP mRNA following treatment with ICA and BMP-2. *p < 0.5, **p < 0.1.

To further investigate the effect of ICA on genes associated with osteoblast proliferation, the mRNA levels of *BMP2, OPG*, and *ALP* genes were determined in hFOB1.19 cells, which were treated with dose-dependent ICA or BMP2. Total RNA of cell samples was extracted and agarose gel electrophoresis confirmed the integrity of total RNA (**Figure 6A**). Consistent with our western blot analysis, we observed that treatment with ICA (10–30 μg/ml) significantly upregulates *BMP-2* and *ALP* mRNA expression, and 20 μg/ml of ICA is the maximal effective concentration, that was equivalent to 50 μg/ml BMP-2 (**Figures 6B, D**). In addition, ICA was capable of upregulating *OPG* mRNA expression within the range of 20 to 30 μg/ml, and 30 μg/ml of ICA is the maximal effective concentration (**Figure 6C**). It worth noting that high dose of ICA (50 μg/ml) did not exert an increase effect on the expression of *BMP2, OPG*, and *ALP* mRNA (**Figure 3**). These results suggested that ICA increases the mRNA levels of *BMP2, OPG*, and *ALP* genes.

### Observational Indices

**Figure 4:**
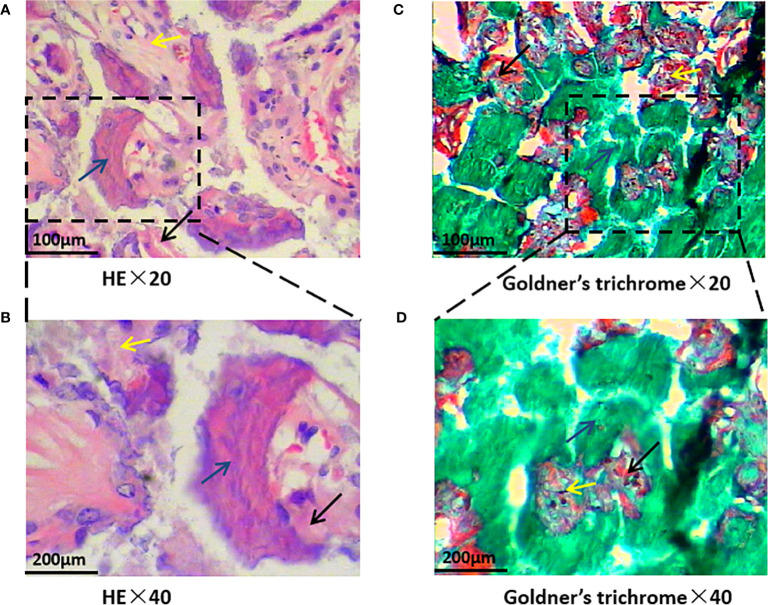
Bone formation by hematoxylin-eosin (HE) and Goldner’s trichrome staining. Black arrows indicate newly mineralized bone, blue arrows indicate osteoidtissues, and yellow arrows indicate the scaffold. HE staining is shown on the left and Goldner’s trichrome staining is shown on the right. **(A, B** showed HE stain, **C, D** showed Goldner’s trichrome stain, and magnify 20 or 40 times).

**Figure 5:**
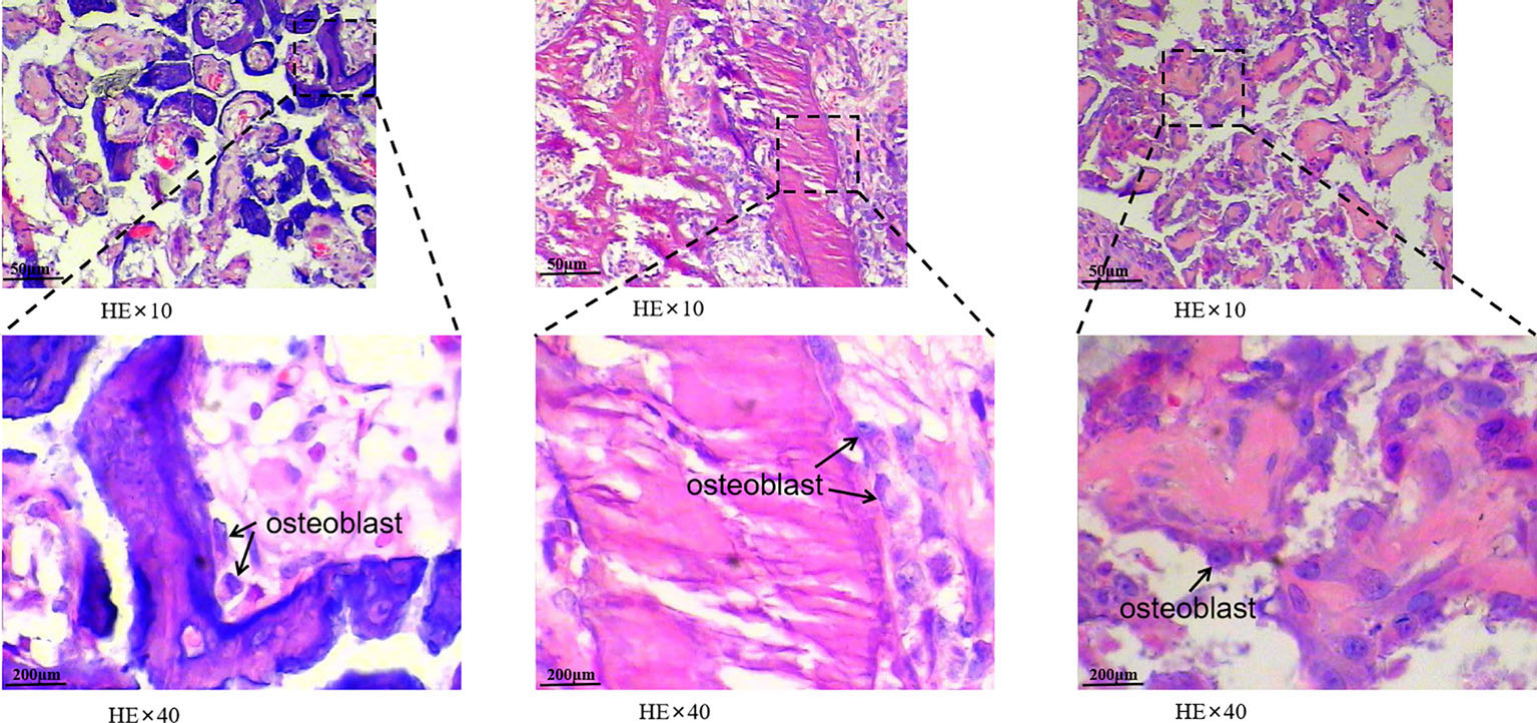
The morphology of cells in bone tissue was observed. The black arrows under a 40-fold light microscope indicate osteoblasts are found in new bone tissue. The osteoblasts in this image are either activated by injury to repair replacement cells or enlarged.

HE staining revealed that trabecular bone and cortical bone were stained light red or pink, collagen fibers stained pale pink, and osteoblasts and osteocytes stained purple-blue. Goldner’s trichrome staining revealed that osteoblasts were stained orange, osteoid tissues stained purplish red, the newly mineralized bone stained blue-green or bright blue, and the mature bone stained green. As the nHAC/PLA scaffold contained calcium salt, it was stained consistent with the osteoid (**Figure 4**). Goldner’s trichrome stain revealed brighter colors than HE stain, and had different results in bone tissues at different mineralization stages, and more osteoblasts have been observed (**Figure 5**).

**Figure 6:**
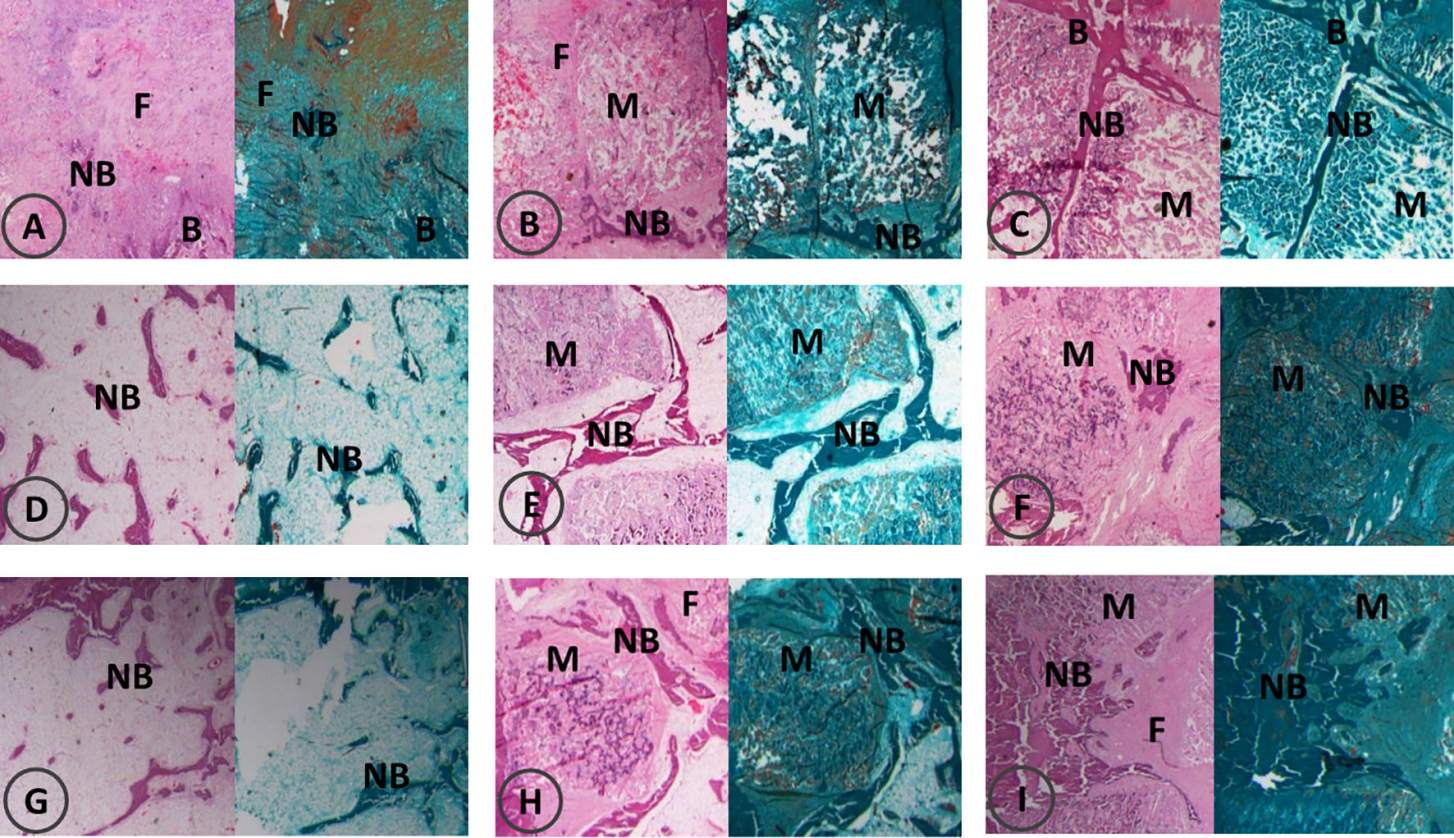
Bone formation by HE and Goldner’s trichrome staining at 4, 8, 12 weeks post-operation. F, fiber; NB, new bone; B, bone; M, materials. HE and Goldner’s trichrome staining at 4 weeks post-operation **(A–C)**; HE and Goldner’s trichrome staining at 8 weeks post-operation **(D–F)**; HE and Goldner’s trichrome staining at 12 weeks post-operation **(G–I)**. In each image, HE staining is shown on the left and Goldner’s trichrome staining is shown on the right; control groups **(A, D, G)**, ICA groups **(B, E, H)**, BMP groups **(C, F, I)**.

At 4 weeks post-operation, HE staining and Goldner’s trichrome staining showed that there was a large amount of fibrous tissue and few new bone formation with abundant fibroblasts in the control group. In the ICA group, new bone strips were distributed at the edge of the material, and the material particles were discontinuously wrapped with new bone or osteoid tissues, and in the BMP-2 group, new bone strips were distributed among the material particles, and new bone mass increased around the material particles (**Figures 6A–C**).

In the control group, a small amount of new bone tissue was observed in the center of the defect at eight weeks post-operation, and the bone mass increased significantly compared with that at four weeks post-operation. Bone strips were interconnected in the ICA group where the material particles were replaced a little by bone tissue. In the BMP-2 group, flaky bone tissues formed and the material particles were completely replaced by bone tissues with an increase in bone maturity (**Figures 6D–F**).

At 12 weeks post-operation, there were bone strips in the center of the defect in the control group, with no obvious increase in bone mass. In the ICA group, there were elongated bone tissues between the material particles. A part of the material degraded and was completely replaced by new bone tissue with corresponding contour. Large areas of flaky bone tissue formed in the BMP group, with increased new bone tissue in quantity and maturity. The contour of the new bones was consistent with that of the original material (**Figures 6G–I**).

## Discussion

Bone defects and osteoporosis have always been challenging for clinicians. Since the start of the 21st century, approximately 2.3 billion US dollars of biomaterials have been used for hard tissue repair and substitute materials worldwide. There were 500 to 1.5 million cases of bone transplantation in the United States alone (Greenwald et al., 2001). Traditional Chinese medicine prescriptions or monomers have been increasingly preferred and used to repair bone defects in China, which demonstrates its potential in bone tissue engineering. *Epimedium brevicornum* Maxim has long been one of the most important traditional Chinese medicines for strengthening kidney and bones, and its osteoinductive activity in bone tissue engineering has been confirmed (Zhao et al., 2010; Ma et al., 2011). ICA is the main active ingredient of *Epimedium brevicornum* Maxim, and it is a promising Chinese herb monomer that has been successfully extracted (Wu and Peng, 2018).

Some Chinese scholars have found that ICA can promote osteogenesis and guide bone regeneration; for example, it can promote the proliferation, osteogenic differentiation potential and activity of osteoblasts and periodontal ligament cells, promote the osteogenic differentiation of bone marrow stromal stem cells, and inhibit adipogenic differentiation (Tu et al., 2017; Li et al., 2019). Zhang et al. (2016a) summarized the molecular mechanism and signaling pathway of ICA. Their findings reveal that it can participate in regulation of various signaling pathways in the entire musculoskeletal system. ICA is an effective compound in promoting bone repair and preventing or delaying the progression of GC-associated *ONFH* in rats. This can be explained by its ability to improve the balance between adipogenesis and osteogenesis (Huang et al., 2018). Through the estrogen receptor-mediated pathway, ICA promotes osteoblast differentiation and proliferation and inhibits osteoclast differentiation and metastasis. Using the *BMP-2/Smad4* signaling pathway, ICA can promote bone formation or increase Ca^2+^ concentration and upregulate BMP-2 by inducing expression of *Osterix* (*OSX)*, *RUNX-2*, and *ALP* genes. In addition, ICA can inhibit osteoclastic bone resorption by regulating *OPG/RANKL* signaling pathway (Cao et al., 2012; Liang et al., 2012; Zhang et al., 2016a). Zhang et al.’s study (2017) has shown that ICA promotes bone formation by up-regulating BMP2/Runx2 and *OPG/RANKL* pathways; ICA exhibited osteoplastic properties on osteoblasts that had been inhibited by high doses of vancomycin which is helpful for treatment post-bone infection.

BMP can effectively induce undifferentiated mesenchymal cells to differentiate into osteoblast cell lines, promote the recruitment and differentiation of undifferentiated mesenchymal cells and bone cells, and initiate bone formation. Furthermore, BMP-2 plays a key role in bone formation, growth, and repair (Li et al., 2005; Urist and Strates, 2009). A study by Xu et al. (2016) indicated that BMP-2 can increase Ca^2+^ concentration and promote osteoblast differentiation by regulating intracellular calcium signaling. Costello et al. (2015) increased the formation and integration of osteogenic mineralized bone nodules using *in vitro* BMP-2 treatment, which in turn induced bone formation. Findings from the current study indicate that ICA can promote the proliferation of osteoblasts by promoting the expression of BMP-2 and OPG proteins and upregulating the expression of *BMP-2*, *OPG*, and *ALP* mRNAs, which is consistent with findings of previous studies. Moreover, this study revealed that certain concentrations of ICA have the same osteogenic potential as BMP-2.

With the development of bone tissue engineering and the diversification of construction modes, the strengths of the controlled-release nano-scaffold system have been highlighted. The current research aim is to obtain different degradation rates by combining different materials (Zhu et al., 2015). ICA can better exert its biological activity with a suitable carrier in the body, otherwise high local concentrations and uneven distribution can trigger its quick absorption (Loretz et al., 2007). Some scholars constructed a three-dimensional structure of collagen hydrogel, and they found that *Epimedium brevicornum Maxim* can effectively enhance *BMP-2* and *RUNX-2* expression at *mRNA* and protein levels compared with *OSX* control group (Xie et al., 2015). ICA-containing poly(3-hydroxybutyrate-co-3-hydroxyvalerate) (PHBV) scaffolds constructed *in vitro* can effectively upregulate the transcription of various growth factors (such as BMP-2) and the transcription of extracellular matrices (Xia et al., 2013). Controlled release of ICA can induce osteogenesis, enhance matrix mineralization, and inhibit osteoclast differentiation, thus shortening bone healing time. In another study, a large number of bone cells and bone connections were observed after implantation of nHAC/PLA carrying P-17-BMP-2, indicating bone repair materials loaded with novel short peptides have good osteoinductive and osteoconductive properties (Zhang et al., 2016b; Wu et al., 2018). In this study, the periosteal nHAC/PLA composite carrying ICA or BMP as tissue-engineered bone was successful without significant rejection *in vivo*. The shape of new bone tissue was consistent with the contour of the degraded material, indicating that there is a dynamic balance between material degradation and new bone formation. The addition of ICA or BMP-2 increased osteogenesis relative to the control group, indicating osteoinductive activity of the two composite materials.

The biological organism has a complex environment. As the non-decalcified slice preserves the mineralized structure of the bone tissue intact, it is easy to distinguish between calcified bone and uncalcified bone using special staining techniques. The Goldner’s trichrome staining was performed on non-decalcified sections, whereas HE staining was performed on decalcified sections. In the Goldner’s trichrome stain, different colors of osteoblasts, osteoid, mineralized bone, and various stages of bone mineralization were significantly compared. HE staining is a simple, convenient, and low-cost method. The combination of HE staining and Goldner’s trichrome staining can compensate for their lack of coloration to obtain comprehensive information on osseointegration and bone remodeling. In this study, there was a dark coloration in Goldner’s trichrome stain. The possible cause may be related to staining time or a low concentration of light green SF yellowish. We introduced ICA or BMP-2 into the pores of the nHAC/PLA materials by external vacuum adsorption, but this method has many limitations, such as insufficient mixing, no objective evaluation, and unclear controllability. Therefore, further exploration of the preparation of composite materials is warranted.

In this study, it was found that *in vivo* animal experiments showed that in the process of bone reconstruction in the early defect area, new bone formation was the main process, and osteoclasts increased in the process of bone remodeling and maturation 1 month later, osteogenesis and osteoclast were carried out at the same time. OPG is the main protein expression in the process of osteogenesis. The correlation of *OPG-RANKL-RANK* expression has been confirmed by related literatures at home and abroad, and the increase or overexpression of OPG may inhibit osteoclast and bone resorption. Some related genes of osteoclasts, such as *Runx2, osterix, BSP*, have also been reported. Therefore, OPG, ALP, and BMP are selected as the main observation indexes in this experiment. According to the previous research results, it is speculated that these genes have certain correlation. The expression of rank may increase correspondingly, while the expression of rank will decrease. Other osteoclast related genes such as *Runx2, osterix*, and *BSP* are still unknown. The specific mechanism of action needs to be verified by further experiments later. This study chose to analyze the expression of *BMP-2*, *OPG*, and *ALP*, as these are the representative genes involved in osteoclast differentiation, osteoclastogenesis, and osteoid mineralization, respectively. Although other osteoclast-related genes such as *Runx2*, *Osterix*, *BSP*, and *RANKL* play important roles in osteoclastogenesis, it is interesting to determine the effect of BMP-2 and ICA on the expression levels of these genes. This study is not yet clear about the rationale behind the combination of BMP2 and ICA as well as the single action, and further analysis will be carried out in the next experiments. Reports have shown that BMP-2 induces *RANKL* expression and regulates *Osterix* through *Msx2* and *Runx2* during osteoblast differentiation in the mesenchymal cell lines C3H10T1/2 and C2C12 (Matsubara et al., 2008; Usui et al., 2008). ICA upregulated the expression of *Runx2 mRNA* in the osteoblastic cell line MC3T3-E1 and treatment with ICA decreased the levels of *RANK* and *RANKL* in human SW1353 chondrosarcoma cells (Zhao et al., 2008; Wang et al., 2014). However, the role of ICA in regulating other osteoclast-related genes including *Osterix*, *BSP*, and *RANKL* remain largely unknown, which is worth further investigation in our future studies. In this study, we attempted to construct the nHAC/PLA composite carrying ICA or BMP-2 with controllable bone conductivity and osteoinductivity, and this composite was expected to be an ideal material for bone repair. This study also explored a new approach to repairing bone defects using additional active monomers of Chinese herbs, providing a new direction for bone defect repair and bone disease treatments.

## Data Availability Statement

The raw data supporting the conclusions of this article will be made available by the authors, without undue reservation, to any qualified researcher.

## Ethics Statement

This animal study was reviewed and approved by the Laboratory Animal Ethics Committee of Guangzhou University of Traditional Chinese Medicine.

## Author Contributions

LX was in charge of the project, mainly responsible for experimental design and data analysis. YL and CY were responsible for the animal experiments. GW was responsible for data processing. NL and YX were responsible for the cell experiments. XJ was in charge of the experiments and evaluation of the results. LX wrote the manuscript. XJ reviewed the article.

## Funding

This study was supported by the research project of The First Affiliated Hospital of Guangzhou University of Chinese Medicine and Medical Research Fund of Guangdong Province (A2016551). The funding body played no role in the study design, in the collection, analysis, and interpretation of data, the writing of the paper, or the decision to submit the paper for publication.

## Conflict of Interest

The authors declare that the research was conducted in the absence of any commercial or financial relationships that could be construed as a potential conflict of interest.

## Abbreviations

ALP, Alkaline phosphatase; BMP-2, Bone morphogenetic protein-2; Ct value, Cycle threshold; ECL, Electrochemiluminescence; MTT, 3-(4,5-dimethylthiazol-2-yl)-2,5-diphenyltetrazolium bromide; nHAC/PLA, Nano-hydroxyapatite/collagen/polylactic acid; OPG, Osteoprotegerin; PHBV, Poly(3-hydroxybutyrate-co-3-hydroxyvalerate).
